# Micronutrient Fortification to Improve Growth and Health of Maternally HIV-Unexposed and Exposed Zambian Infants: A Randomised Controlled Trial

**DOI:** 10.1371/journal.pone.0011165

**Published:** 2010-06-17

**Authors:** The Chilenje Infant Growth, Nutrition and Infection (CIGNIS) Study Team

**Affiliations:** Tulane University, United States of America

## Abstract

**Background:**

The period of complementary feeding, starting around 6 months of age, is a time of high risk for growth faltering and morbidity. Low micronutrient density of locally available foods is a common problem in low income countries. Children of HIV-infected women are especially vulnerable. Although antiretroviral prophylaxis can reduce breast milk HIV transmission in early infancy, there are no clear feeding guidelines for after 6 months. There is a need for acceptable, feasible, affordable, sustainable and safe (AFASS by WHO terminology) foods for both HIV-exposed and unexposed children after 6 months of age.

**Methods and Findings:**

We conducted in Lusaka, Zambia, a randomised double-blind trial of two locally made infant foods: porridges made of flour composed of maize, beans, bambaranuts and groundnuts. One flour contained a basal and the other a rich level of micronutrient fortification. Infants (n = 743) aged 6 months were randomised to receive either regime for 12 months. The primary outcome was stunting (length-for-age Z<−2) at age 18 months. No significant differences were seen between trial arms overall in proportion stunted at 18 months (adjusted odds ratio 0.87; 95% CI 0.50, 1.53; P = 0.63), mean length-for-age Z score, or rate of hospital referral or death. Among children of HIV-infected mothers who breastfed <6 months (53% of HIV-infected mothers), the richly-fortified porridge increased length-for-age and reduced stunting (adjusted odds ratio 0.17; 95% CI 0.04, 0.84; P = 0.03). Rich fortification improved iron status at 18 months as measured by hemoglobin, ferritin and serum transferrin receptors.

**Conclusions:**

In the whole study population, the rich micronutrient fortification did not reduce stunting or hospital referral but did improve iron status and reduce anemia. Importantly, in the infants of HIV-infected mothers who stopped breastfeeding before 6 months, the rich fortification improved linear growth. Provision of such fortified foods may benefit health of these high risk infants.

**Trial registration:**

Controlled-Trials.com ISRCTN37460449

## Introduction

The period of complementary feeding is a period of high risk for growth faltering which may result in permanent stunting and its adverse health consequences. [1] Although there has been some progress in promoting and supporting exclusive breastfeeding, [2] programmes addressing complementary feeding [3] in the second half of infancy and early childhood have yet to meet with similar success.

Infant and young child feeding practices are further complicated in HIV-endemic sub-Saharan Africa. HIV-exposed infants, even if not themselves infected, are at increased risk of poor growth and death; mechanisms are unclear but likely involve nutrition [4]. HIV-infected women are advised to exclusively breastfeed their infants unless they have access to replacement foods which are acceptable, feasible, affordable, sustainable and safe (AFASS; [5]). Recent studies have shown that providing antiretroviral drugs to women or infants for the first few months after delivery can reduce breast milk transmission of HIV [6]. However, it remains unclear when to stop these antiretrovirals in women who do not yet need them for their own health and when HIV-infected women should stop breastfeeding. Given the continuing transmission risk throughout breastfeeding [6], many HIV-infected women wish to stop early, especially if their infant is HIV-negative at around 6 months old. Furthermore, after about 6 months, other foods need to be introduced to the infant's diet, whether or not the mother continues to breastfeed [3]. Unfortunately, in much of sub-Saharan Africa available foods for infants are grossly inadequate whether they are fed as complementary foods or as replacement foods in the absence of breastfeeding [4], [7], [8].

A major problem with available infant foods is low micronutrient density. [9] Micronutrient deficiencies are common in Zambia. [10], [11] Micronutrient-rich foods are often expensive and fortification of widely consumed foods may be a more effective intervention to improve micronutrient status than promotion of consumption of foods naturally rich in micronutrients. Furthermore, central fortification requires less active consumer participation than micronutrient supplementation and is not associated with adverse effects seen with some supplements, notably iron [12]. Zambia has a track record of successful implementation of universal fortification of sugar with vitamin A and salt with iodine. [10], [13] There have been plans to fortify the staple, maize, with several micronutrients. [10] However, the fortificant levels for general consumption are unlikely to meet the micronutrient needs of young children who have fast growth rates yet low food intakes. The requirements for many micronutrients in children aged 6 to 24 months are not well known and have often been extrapolated from levels consumed by healthy, older children.[9] As a result, some estimated requirements may be in excess of the true requirements and it may be unnecessarily costly to meet them. A recent meta-analysis [14] concluded that multiple micronutrient interventions had modest benefits for linear growth among primarily breastfed infants and young children but the trials have not addressed non-breastfed infants.

We have conducted a randomised, double-blind, controlled trial of the efficacy of two locally produced complementary/replacement foods, fortified with different levels of micronutrients, for infants aged 6 to 18 months. To our knowledge, this is the first study investigating AFASS foods for infants of HIV-infected mothers after 6 months, is the largest trial of multiple micronutrients for children of this age [14], and is a relatively rare double-blind, controlled trial of a food-based intervention. Many previous studies have used supplementation approaches which are generally easier to run and control at the trial stage but use interventions which may be less easy to implement sustainably than fortification approaches.

## Methods

### Study design

The trial is registered as ISRCTN37460449 (www.controlled-trials.com/mrct). The protocol for this trial and supporting CONSORT checklist are available as supporting information; see Checklist S1 and Protocol S1. Children were randomised to one of two locally produced porridges at age 6 months, and were followed monthly for 12 months. The primary outcome was the prevalence of stunting at 18 months, chosen since stunting begins in early infancy, is the most common form of malnutrition in young children, is largely irreversible, and is associated with long term poor health and development. The key secondary outcome was hospital referral or death.

### Study population and recruitment

The trial was conducted in Chilenje, Lusaka, Zambia, from October 2005 to July 2009. Infants were recruited by informing mothers attending the child health section of the government clinic on the same site as the project clinic. As over 90% of Lusaka women bring their infants to the clinic for monthly weighing or standard early vaccinations, [15] the population hearing about the study was representative of the area. According to Zambian government policy, all children receive the following vitamin A supplements at these ages: 0–5 months and not breastfeeding 15 mg, all children 6–11 months 30 mg, all children 12–59 months 60 mg. Study staff arranged for the 6, 12 and 18 month doses for study children.

Infants were eligible if they were 6 months +/−2 weeks old and in generally good health and their mothers gave written consent to attend monthly clinic visits for the next year, feed their infants the allocated porridge, have their infant undergo project tests including blood and urine sampling, and have their child tested for HIV at 18 months old. Knowledge of maternal HIV status was not a requirement for joining the study but 90% of women knew their status from antenatal HIV antibody testing in the government health service using the same algorithm described below for project testing.

### Randomisation

Randomisation was in blocks of 20; the code was held by the project Data Safety and Monitoring Board (DSMB). One kg bags of porridge flour were labelled with the participant ID number. At the recommendation of the DSMB, the randomisation and labelling process was audited halfway through the study by an independent observer and found to be robust, accurate and able to maintain blinding of other study staff and participants.

Infants were allotted to porridges by sequential numbers on recruitment. Twins (n = 13 pairs) were randomised to the same diet and included in analyses as separate individuals. There was one serious protocol violation: during May – June 2007 one porridge ran out but staff continued to recruit to the other treatment; the children who were thus not appropriately randomised were followed for ethical reasons but are not included in the present analyses.

### Study porridges

The two flours, based on maize, beans, bambaranuts and groundnuts, were similar in bulk ingredients and macronutrient content but differed in micronutrient content (Table 1). The flours were mixed with water to make porridges for infant feeding, similar in consistency and appearance to those in current local use. In pilot work, porridges were found to be acceptable [16] to the local population and to displace other micronutrient-poor complementary or family foods but not to displace breast milk intake [17]. The energy density of the porridge prepared according to package directions (equivalent to 30g blended in 250 ml water) was 0.76 kcal/g; [18] protein represented 16% of energy. The richly fortified porridge contained micronutrients to meet the World Health Organization (WHO) estimated needs [9] for infants aged 9–11 months with low breast milk intake, assuming an intake of 50g flour/day. We did not include iodine in the micronutrient premix since Zambian salt is fortified with iodine. The basal porridge had micronutrient levels which would be produced without any additional fortificants once maize in Zambia is fortified at the planned level. [10], [19] Micronutrient premixes for both flours were prepared by DSM (Isando, South Africa). Flour was mixed and processed by extrusion at Quality Commodities Ltd, Lusaka. Porridge flours were stored for up to 6 months in a cool, well-ventilated room.

**Table 1:** Composition of the trial porridge flours.

	Basal fortification (per kg flour)^1^	Rich fortification (per kg flour)^2^
maize	650 g	615 g
groundnuts	150 g	150 g
bambaranuts	50 g	50 g
beans (white and yellow)	150 g	150 g
energy	4200 kcal	4140 kcal
protein	15%	15%
fat	12%	12%
vitamin A	0.65 RE	6.5 RE
vitamin C		2.0 g
vitamin D		0.1 mg
thiamin (mononitrate)	1.3 mg	9 mg
riboflavin	1.6 mg	11.2 mg
niacin (niacinamide)	13 mg	140 mg
pyridoxine (HCl)	1.6 mg	8.6 mg
folate	0.65 mg	2.21 mg
B12	3.25 µg	9.75 µg
pantothenic acid		40.3 mg
magnesium (oxide)		943 mg
iron (ferrous fumarate)^3^	6.5 mg	250 mg
zinc (oxide)^3^	9.75 mg	200 mg
copper (gluconate)		3.2 mg
manganese (sulfate monohydrate)		12.0 mg
selenium (sodium selenite)		0.2 mg
calcium (CaH(PO_4_)*2H_2_O)^3^		6.8 g
phosphorus (CaH(PO_4_)*2H_2_O)		5.3 g

1 Based on estimated amounts of micronutrients which would be available from 50 g porridge flour/day made with maize fortified at levels planned nationally for Zambia. Overages (usually 10%) added to allow for losses during processing.

2 Based on total estimated micronutrient needs of infants aged 9–11 months minus amounts expected from low breast milk intakes [9] and assuming intake of 50g porridge flour/day. Exceptions to the micronutrient levels are that vitamin C was increased to compensate for low iron bioavailability and zinc was based on Recommended Daily Allowance set by the International Zinc Consultative Group. [39] Overages (usually 10%) added to allow for losses during processing.

3 Phytate was analysed in two batches of each flour and averaged 5.8 g/kg. This translated into phytate∶zinc molar ratios of ∼19 in the basal flour and ∼3.3 in the richly fortified flour and phytate∶iron molar ratios of 5.6 and 1.7 in the two flours, respectively.

Each batch of flour was analysed by the National Institute for Scientific and Industrial Research, Lusaka, for macronutrients by proximate analysis and for iron and zinc by flame atomic absorption spectroscopy. [20] All batches contained less than the tolerance levels of aflatoxin ([21]; analyses by Cheetah, Zambia Ltd, Lusaka, or Polucon, Mombasa, Kenya). Several batches were also analysed for phytate by HPLC [22] in the laboratory of RSG at the University of Otago, New Zealand.

### Follow-up and adherence

At recruitment, socio-demographic information was obtained by questionnaire. Weight, adherence, and morbidity were assessed monthly and length and other anthropometry 3-monthly in the clinic. Women were given 4 kg of porridge flour each month and could return for more as required. They were instructed to prepare the porridge according to the directions on the package, using a plastic cup, graduated in mL, supplied by the investigators. Adherence was determined through monthly questioning of mothers as to how much prepared porridge the child had consumed (in mL) within each of the two days prior to the visit and whether consumption on these days was typical. For each month the mean and standard deviation (SD) reported intake of the porridge food provided was calculated for ‘typical’ days. Infants were given adherence scores of 1 (low, <1 SD below the mean), 2 (average, ±1 SD of the mean), or 3 (high, >1 SD above the mean). Missed visits were assigned an adherence value of 1. The values for each month were added in order to calculate a cumulative adherence score.

### Anthropometry

Anthropometric measurements were taken using calibrated equipment and standardized techniques, [23] with children nude or wearing a diaper. Maternal weight and height were measured at recruitment using, respectively, a digital scale (to 100 g) and wall-mounted flexible tape (to 1 mm). Infant weight was measured on a digital balance (to 10 g) and length on a length board to 1 mm (anthropometry equipment from Chasmor Ltd, London, UK). Staff conducting anthropometry were trained by RSG and AM using a repeat-measures protocol until they reached an acceptable level of proficiency. [24] Measurement quality was maintained by achieving acceptable ranges for both the differences in measurements between the designated criterion anthropometrist (MC) and the other anthropometrists [24] and for the total technical error of each measurement. [25]

All anthropometric measurements were done in triplicate and the median used in analyses. Standard deviation, Z, scores were calculated using the WHO growth reference data [26] and the child's exact age at measurement. Stunting was considered as length/age Z<−2.

### Morbidity assessments

At all scheduled and unscheduled visits the clinical officer (JS) examined children and diagnosed and treated them according to the Integrated Management of Childhood Illness. [27] Basic care plus prescription of antibiotics or antimalarials were possible within the study clinic. Patients were referred either to Chilenje main clinic, where limited services were available, or to University Teaching Hospital (UTH), the local tertiary facility, only when exhibiting danger signs (unable to drink or breastfeed, severe vomiting, convulsions, respiratory distress, lethargic or unconscious), when requiring surgery, or when repeatedly presenting with an intractable illness which required specialist consultation. Diagnoses and treatments in hospital were determined by doctors not connected with the CIGNIS study; information about these was available to the project. Referral to hospital or specialist clinic services was a trial outcome.

Classification and treatment of illness was based on local practice. If malaria was suspected from history or clinical presentation, a blood slide was taken and malaria was diagnosed if parasites were present. Further investigations were carried out in UTH for fevers without parasites, including lumbar puncture in cases of suspected meningitis. Diarrhea was defined as at least 3 loose stools or one bulky watery stool in the past 24 hours. Pneumonia was defined as history of cough or difficulty breathing plus rapid breathing (≥50 breaths/minute for infants and ≥40 breaths/minute for older children) with or without subcostal recession.

### HIV testing

Serum samples from all children at 18 months were tested for antibodies to HIV. A serial testing algorithm was used. Samples were first tested using Determine HIV 1/2 (Inverness Medical, Japan). If negative, the result is taken to be negative. If positive, a second test, Unigold HIV 1/2 (Trinity Biotech plc, Ireland), was used. If positive, the final result is positive but if negative, i.e. discordant, a third test SD-Bioline HIV 1/2 (Standard Diagnostics, Korea) was used and its result was taken to be the final result.

### Iron status

Three indicators of iron status were measured at baseline and 18 months of age. Hemoglobin was measured in fingerprick blood in by Hemocue (Dronfield, UK). Venous blood was collected in plain vacutainers, sera separated at UTH within 2 hours, aliquoted and stored at −80°C. Samples were transported to London for serum ferritin and transferrin receptor (TRFR) assays. Ferritin was measured by ELISA [28] using antibodies from Insight Biotechnology Ltd (Wembley, UK) and Invitrogen Ltd (Paisley, UK) with standards from Dade Behring (Milton Keynes, UK). The interassay coefficient of variation (CV) for the standard (National Institute of Biological Standards and Control, Potters Bar, UK) was 12%. TRFR was measured by ELISA using a commercial kit (RAMCO, ATI Atlas, Chichester, UK). The interassay CVs for the high and low quality control sera were, respectively, 6.9% and 9.5%. For both assays, external quality control standards were within expected ranges.

### Data management and statistics

Data were double-entered and verified in Access. Range and logical checks were run in Access and in Stata (Statacorp, College Station, TX, USA). Stata version 10 was used for all analyses. The analysis plan was finalised before the trial was unblinded.

The impact of richly-fortified porridge on stunting at 18 months (the primary endpoint) was estimated using odds ratios (OR) and 95% confidence intervals (CI) obtained by logistic regression. Length-for-age Z score was also analysed as a continuous variable with differences by linear regression. Severe morbidity, defined as hospital referral or death, was evaluated using Kaplan-Meier curves. The impact of the richly-fortified porridge on severe morbidity was estimated using hazard ratios (HR) and 95% CI obtained by Cox regression with robust standard errors to account for repeated referrals within children. Hospital referrals for planned surgeries (5 circumcisions, 4 hernias, and 4 other congenital abnormalities) were excluded from the analysis.

All analyses were stratified by maternal HIV status. The primary analyses of both outcomes were intention to treat and unadjusted. Maternal education, and socioeconomic status were considered potential confounders, and were adjusted for in secondary analyses. Socioeconomic status was measured using an asset index, created by combining data on possessions and housing characteristics using principal component analysis. [29] Since determining optimal feeding of HIV-exposed infants was a key aim of the study, analyses of children of HIV-infected mothers were stratified by breastfeeding duration.

### Sample size

The sample size calculations assumed a stunting prevalence at 18 months of 40% [30] among children receiving the basal porridge. Allowing for a 15% loss to follow up, a sample size of 400 per group was estimated to provide >90% power to detect a 30% reduction in the prevalence of stunting, and >80% power to detect a 25% reduction (alpha = 0.05). The DSMB reviewed the results twice (September 2007 and May 2008) and, after noting the prevalence of stunting was lower than anticipated and determining that an increase in sample size was financially and logistically impossible, recommended stopping recruitment in mid-July 2008.

### Ethics

The study was approved by the ethics committees of the University of Zambia and the London School of Hygiene and Tropical Medicine. Mothers gave written informed consent. Project staff referred participants who were ill to local medical services and followed up treatments within these services. Neither funding source (the Bill and Melinda Gates Foundation and DSM South Africa) was involved in conduct, analysis or interpretation of the study.

## Results

**Figure 1:**
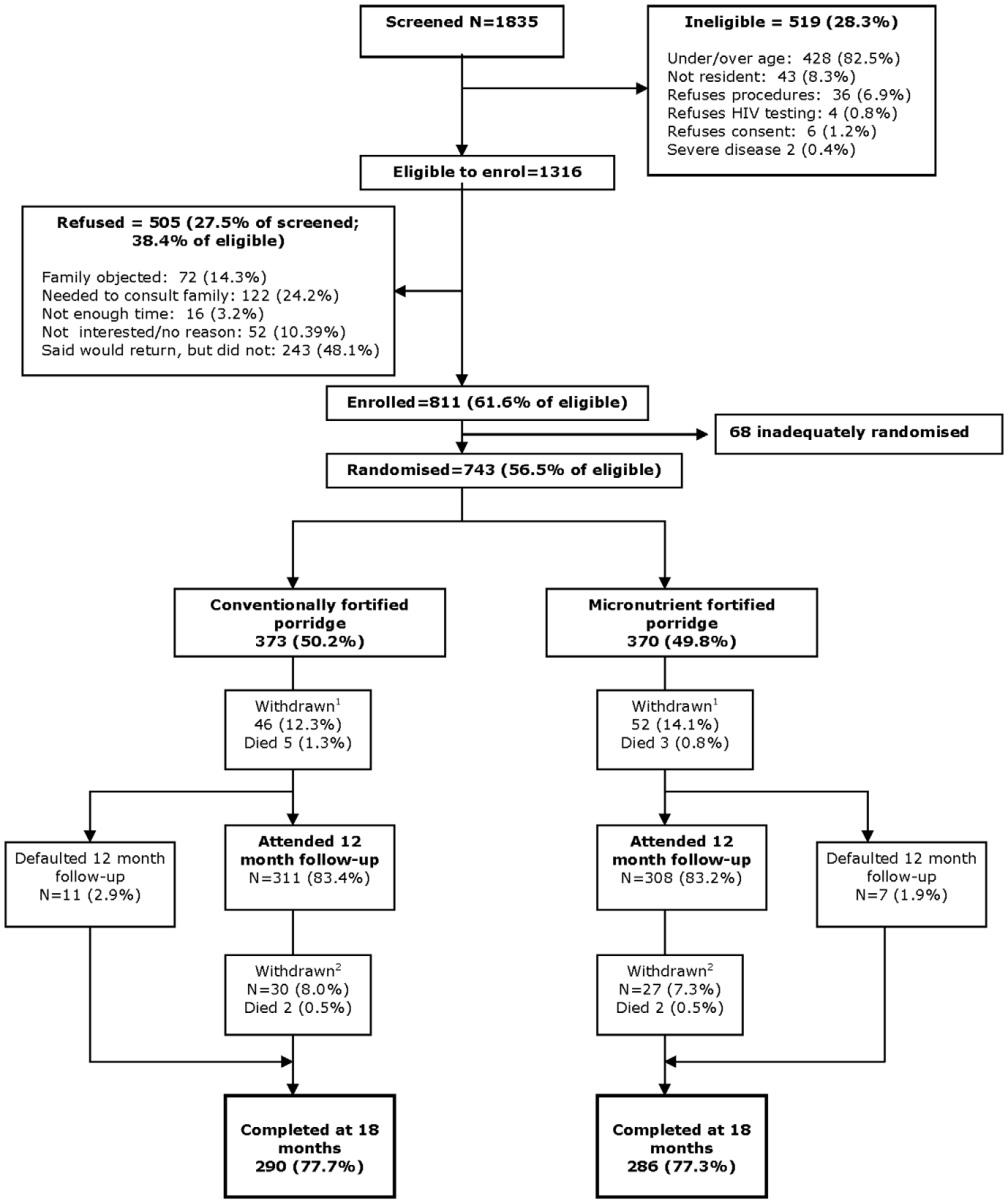
Flow diagram of participants through the study. All recruited children were included in the analysis of hospital referral and death. Two children in the richly fortified group who were seen at 18 months had missing length data at that visit and are not included in the analysis of stunting. ^1^Reasons for withdrawal up to 12m: basal porridge (N = 46): moved away (15), family against (10), problems with porridge (2), would not say/other (19); richly fortified porridge (N = 52): moved away (18), family against (10), would not say/other (24). ^2^Reasons for withdrawal from 12–18m: basal porridge (N = 30): moved away (13), family against (1), problems with porridge (2), would not say/other (14); richly fortified porridge (N = 27): moved away (8), family against (1), problems with porridge (1), would not say/other (17).

A total of 743 infants were recruited and randomised (Figure 1). There was a large number of refusals, mostly by women going away to think about it and not returning.

The trial arms were generally balanced at baseline (Table 2). Seventy percent of mothers tested HIV-negative antenatally, 21% tested HIV-positive and 9% did not know or did not wish to disclose their status. HIV-infected women were older than uninfected women (28.5 (SD 5.4) versus 25.5 (SD 5.7) years) and were of slightly lower educational level and socioeconomic status, although the differences were not significant (data not shown). The majority (96%) of HIV-uninfected women were breastfeeding at recruitment, but 38 (24%) of HIV-infected women did not initiate breastfeeding, and an additional 50 (29%) stopped before the infant was 6 months old.

**Table 2:** Description of the study population at recruitment.

	Basal porridge	Richly fortified porridge
**N**	373	370
**Maternal and demographic characteristics**		
Maternal antenatal HIV status^1^		
Negative	261 (70%)	258 (70%)
Positive	79 (21%)	78 (21%)
Unknown	33 (9%)	34 (9%)
Mother's age (years)	26.0 (SD 5.7)	26.4 (SD 5.8)
Maternal body mass index (kg/m^2^)		
<18.5	29 (8%)	29 (8%)
18.5–25	225 (61%)	210 (57%)
25–30	76 (20%)	92 (25%)
>30	42 (11%)	39 (11%)
Mother's education		
primary or less	123 (33%)	129 (35%)
secondary	157 (42%)	127 (34%)
college/university	93 (25%)	114 (31%)
Mother's occupation		
housewife	214 (57%)	181 (49%)
salaried employee	61 (16%)	86 (23%)
self-employed	39 (11%)	31 (8%)
other	59 (16%)	72 (20%)
Marital status		
married	278 (75%)	279 (75%)
single	79 (21%)	72 (20%)
divorced, separated, widowed	16 (4%)	19 (5%)
Tertiles of socioeconomic status		
low	127 (34%)	123 (33%)
middle	151 (40%)	136 (37%)
high	95 (25%)	111 (30%)
**Infant characteristics**		
Sex (#, % F)	182 (49%)	204 (55%)
Birth weight (kg)	3.05 (SD 0.50)	3.05 (SD 0.46)
Weight at 6 months (kg)	7.27 (SD 1.05)	7.28 (SD 1.10)
Length at 6 months (cm)	64.9 (SD 2.3)	64.9 (SD 2.6)
Proportion stunted (length for age <−2 Z)	51 (14%)	40 (11%)
Blood haemoglobin <105 g/L	140 (38%)	149 (41%)
Breastfeeding duration:		
never	22 (6%)	21 (6%)
<6 months	38 (10%)	29 (8%)
breastfeeding at recruitment	313 (84%)	320 (87%)

1 Six infants of mothers HIV-uninfected antenatally and one of an HIV-unknown mother tested HIV-infected by 18 months old.

There was a 23% loss to follow-up before the infant reached 18 months. All women were contacted by study personnel to ask why they discontinued in the study. The reasons did not differ by treatment group and were: child death (12, 7%), did not want to give a reason (56, 34%), family moved from the study area (54, 32%), family opposition (22, 13%), reported problems with the study food (5, 3%), other reasons (18, 11%). There were more HIV-uninfected women among those lost to follow-up (77%) than among those who completed (68%) but these groups were otherwise very similar.

The amount of porridge consumed increased with age from ∼270 mls/d (∼19 g flour/d) at 7 months to ∼350 mls/d (∼25 g flour/d) from 11 to 18 months. Neither the number of visits attended nor adherence differed between treatment groups (OR (95% CI) for proportion of visits with high adherence in the richly-fortified compared with the basal diet: 0.79 (0.61, 1.04)). The proportion of visits with high adherence was significantly greater among children of HIV-infected women compared with HIV-uninfected women (OR (95% CI): 1.81 (1.35, 2.41)) and was significantly lower among children currently breastfed compared with those not breastfed (OR (95% CI): 0.56 (0.44, 0.70)), those in the highest socioeconomic status quintile compared with the lowest (OR (95% CI): 0.51 (0.34, 0.76)) and those whose mothers had college education compared with primary education or less (OR (95% CI): 0.63 (0.44, 0.91)).

The richly-fortified porridge increased haemoglobin between 6 and 18 months, and prevented the deterioration in iron status assessed by serum ferritin or transferrin receptors seen in the basal porridge group (Table 3). Similar effects were seen for children of HIV-infected and uninfected and in those breastfed or not at recruitment (data not shown). Adherence was associated with these improvements in iron status in the richly-fortified porridge group (data not shown).

**Table 3:** Indicators of iron status at 6 and 18 months of age among those who completed to 18 months^1^.

	6 Months		18 Months			
	Basal porridge	Richly fortified porridge	Basal porridge	Richly fortified porridge	Unadjusted regression coefficient or Odds ratio (95% CI)	P-value
Haemoglobin:						
N	285	278	286	282		
mean (SD) g/L	108 (12)	107 (14)	107 (13)	112 (11)	5.53 (3.55, 7.51)	<0.001
n (%)<105 g/L	103 (36%)	115 (41%)	114 (40%)	63 (22%)	0.43 (0.30, 0.63)	<0.001
Serum Ferritin:						
N	270	264	266	262		
median (p25, p75) µg/L	13. 2 (6.3, 24.8)	15.2 (7.8, 26.1)	8.7 (5.3, 14.2)	13.6 (9.1, 23.7)	0.49 (0.36, 0.62)	<0.001
n (%)<10 µg/L	98 (36%)	80 (30%)	154 (58%)	79 (30%)	0.31 (0.22, 0.45)	<0.001
Serum transferrin receptor:						
N	267	263	271	252		
median (p25, p75) µg/L	8.1 (6.7, 10.1)	7.9 (6.6, 9.6)	9.6 (7.9, 12.5)	7.9 (6.6, 9.6)	–0.27 (–0.33, −0.20)	<0.001
n (%)>11 mg/L	48 (18%)	40 (15%)	92 (34%)	35 (14%)	0.31 (0.20, 0.49)	<0.001

1 Serum ferritin and transferrin receptor log-transformed for analysis; medians, 25^th^ and 75^th^ percentiles presented. Unadjusted regression coefficients and odds ratios represent overall differences between treatment arms.

Children of HIV-infected women had a higher prevalence of stunting at 18 months (33/133, 29%) than children of HIV-uninfected women (71/390, 18%; OR 1.80; 95% CI 1.14, 2.83; P = 0.03). Breastfeeding duration was not associated with stunting at 18 months. Children in the lowest socioeconomic group, those with mothers with only primary education, and those stunted at baseline were more likely to be stunted at 18 months (Table 4). Treatment did not affect stunting or mean length-for-age Z score at 18 months overall, nor when stratified by maternal antenatal HIV status (Table 5). After controlling for baseline length-for-age Z score, socioeconomic status and maternal education, indices of linear growth were significantly increased by treatment among children of HIV-infected women breastfed for less than 6 months; higher adherence was associated with greater benefit. Inclusion of only known HIV-uninfected children did not change this result.

**Table 4:** Factors associated with stunting at 18 months.

	N (%)	Unadjusted OR (95% CI)
**Treatment group**		P = 0.85
Basal porridge	60/290 (20.7%)	1
Richly-fortified porridge	57/284 (20.1%)	0.96 (0.64, 1.44)
**Association with other factors**		
Maternal HIV:		P = 0.03
Negative	71/390 (18.2%)	1
Positive	38/133 (28.6%)	1.80 (1.14, 2.83)
Unknown	8/51 (15.7%)	0.84 (0.38, 1.86)
Child HIV:		P = 0.46
Negative	112/555 (20.2%)	1
Positive	4/14 (28.6%)	1.58 (0.49, 5.14)
Breastfeeding duration in HIV negative mothers:		P = 0.02
<12 months	2/41 (4.9%)	1
12–17 months	37/199 (18.6%)	4.45 (1.03, 19.28)
18+ months	32/.150 (21.3%)	5.29 (1.21, 23.08)
Breastfeeding duration in HIV positive mothers:		P = 0.44
Never	8/34 (23.5%)	1
<6 months	15/44 (34.1%)	1.68 (0.61, 4.61)
6+ months	15/55 (27.3%)	1.22 (0.45, 3.28)
Socioeconomic status:		P<0.001
Low	61/185 (33.0%)	1
Middle	38/226 (16.8%)	0.41 (0.26, 0.65)
High	18/163 (11.0%)	0.25 (0.14, 0.45)
Mother education:		P<0.001
Primary or less	63/181 (34.8%)	1
Secondary	38/228 (16.7%)	0.37 (0.24, 0.60)
College/university	16/165 (9.7%)	0.20 (0.11, 0.37)
Stunted at baseline:		P<0.001
No	62/501 (12.4%)	1
Yes	54/72 (75.0%)	21.25 (11.70, 38.55)

**Table 5:** Effect of porridge group on prevalence of stunting (length-for-age Z<−2) and mean length-for-age Z scores at 18 months.^1^

	Basal porridge	Richly fortified porridge	Unadjusted regression coefficient or Odds ratio (95% CI)	p-value	Adjusted^2^ regression coefficient or Odds ratio (95% CI)	p-value
**All children:**						
N	290	284				
n (%) stunting	60 (20.7%)	57 (20.1%)	0.96 (0.64, 1.44)	0.85	0.87 (0.50, 1.53)	0.63
mean (SD) length/age Z	−1.12 (1.11)	−1.05 (1.20)	0.08 (−0.11, 0.27)	0.42	0.04 (−0.07, 0.15)	0.47
**Stratified by maternal HIV status** 3 **:**						
Negative:						
N	194	196				
n (%) stunting	36 (18.6%)	35 (17.9%)	0.95 (0.57, 1.60)	0.86	0.92 (0.45, 1.88)	0.82
mean (SD) length/age Z	−1.07 (1.04)	−0.94 (1.22)	0.14 (−0.09, 0.36)	0.24	0.07 (−0.06, 0.21)	0.30
Positive:						
N	70	63				
n (%) stunting	21 (30.0%)	17 (27.0%)	0.86 (0.41, 1.84)	0.70	0.41 (0.13, 1.26)	0.12
mean (SD) length/age Z	−1.36 (1.25)	−1.38 (1.16)	−0.02 (−0.44, 0.39)	0.91	0.17 (−0.07, 0.42)	0.17
**Mother HIV positive, stratified by** 3 **:**						
Breastfeeding <6 months:						
N	43	35				
n (%) stunting	15 (34.9%)	8 (22.9%)	0.55 (0.20, 1.52)	0.24	0.17 (0.04, 0.85)	0.03
mean (SD) length/age Z	−1.40 (1.34)	−1.23 (1.28)	0.18 (−0.42, 0.77)	0.56	0.40 (0.05, 0.75)	0.02
Breastfeeding ≥6 months:						
N	27	28				
n (%) stunting	6 (22.2%)	9 (32.1%)	1.66 (0.50, 5.53)	0.41	1.10 (0.16, 7.53)	0.92
mean (SD) length/age Z	−1.28 (1.13)	−1.57 (0.97)	−0.29 (−0.86, 0.28)	0.31	−0.04 (−0.38, 0.31)	0.84
**Mother HIV positive, child HIV negative, stratified by** 3 **:**						
Breastfeeding <6 months:						
N	41	32				
n (%) stunting	14 (35.2%)	8 (25.0%)	0.64 (0.23, 1.80)	0.40	0.20 (0.04, 0.98)	0.048
mean (SD) length/age Z	−1.36 (1.29)	−1.21 (1.34)	0.15 (−0.47, 0.77)	0.63	0.44 (0.08, 0.80)	0.02
Breastfeeding ≥6 months:						
N	25	27				
n (%) stunting	5 (20.0%)	9 (33.3%)	2.00 (0.56, 7.09)	0.28	3.61 (0.35, 37.32)	0.28
mean (SD) length/age Z	−1.22 (1.14)	−1.60 (0.98)	−0.39 (−0.98, 0.20)	0.20	−0.20 (−0.55, 0.15)	0.26

1 Two children, both in the richly fortified arm, were missing length data at 18 months.

2 Adjusted for baseline (6 month) length-for-age Z score, asset index score, and maternal education.

3 No subgroup analyses are presented for HIV-unknown mothers because there were mixed reasons for lack of HIV testing. Stratification by breastfeeding duration was done only for HIV-exposed children since this is the group for which an evidence base is required for policy.

Twelve children died, all in hospital: 7 in the basal porridge group and 5 in the richly-fortified porridge group. Causes of death were: 4 pneumonia, 3 diarrhea, 1 tuberculosis, 1 malaria, 1 meningitis, 1 anemia, 1 choking due to epileptic convulsions. Three of those in the basal group and all 5 in the richly-fortified group had HIV-infected mothers. Only one child could be HIV-tested before death and was found positive. Nine infants of mothers HIV-infected antenatally, 6 infants of mothers negative antenatally and two infants whose mother's status was unknown tested positive themselves.

**Figure 2:**
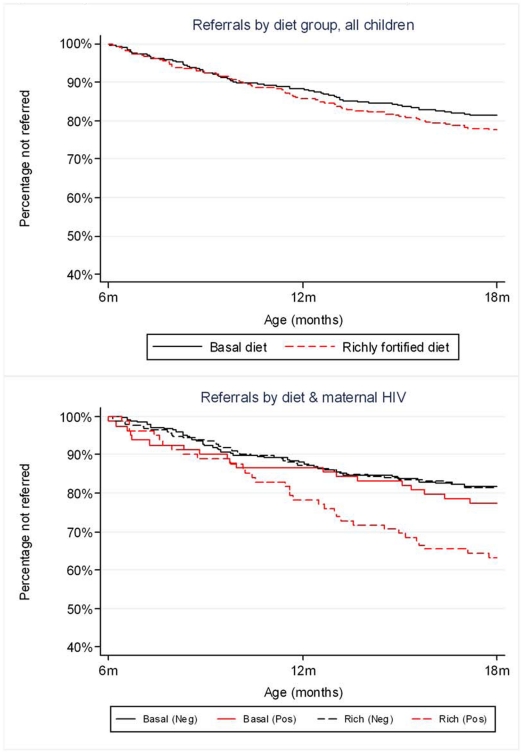
Kaplan-Meier estimates of survivor function for hospital referral or death. All 12 deaths occurred in hospital. Dropouts were censored at the date of last project visit. Admitted children became at risk again on discharge from hospital. In Figure 2b. ‘neg’ = HIV-uninfected mother; ‘pos’ = HIV-infected mother; HIV-unknown women are not included. There were no significant differences by treatment either overall (P = 0.31) or within maternal HIV status groups (P = 0.98 for children of HIV-negative mothers and P = 0.07 for children of HIV-positive mothers).

There were 69 referrals among 64 (17%) children in the basal diet group and 81 referrals among 65 (17%) children in the richly fortified diet group (Table 6). In both groups, 91% of referrals were in-patient admissions. The causes of referral were similar in the two porridge groups except that there were slightly fewer for malaria (HR = 0.63; 95% CI 0.32, 1.24; p = 0.18) and significantly more for pneumonia (HR = 2.68, 95% CI = 1.17–6.11, p = 0.02) in the richly-fortified group. Trial arm had no significant effect overall on the rate of total referrals (Table 7 and Figure 2). Similar results were seen if time to first referral only was analysed (data not shown). There was a trend towards increased referrals among children of HIV-infected mothers in the richly-fortified group, although this did not reach statistical significance. This trend appeared to be mostly due to effects in children who were HIV-infected, but appeared independent of breastfeeding duration.

**Table 6:** Hospital referrals and deaths by porridge group.

	Basal diet	Richly fortified diet
N	373	370
Deaths (% of all children)	7 (1.9%)	5 (1.4%)
Known HIV positive (% of all children)	9 (2.4%)	8 (2.2%)
Children with 1 or more referrals:	64 (17.2%)	65 (17.6%)
Total number of referrals^1^	69	81
Diagnoses (total referrals, % of referrals; % of all children^2^)		
All children:		
Malaria	24 (34.8%; 5.9%)	15 (18.5%; 3.8%)
Acute diarrhea	16 (23.2%; 4.3%)	17 (21.0%; 4.3%)
Pneumonia	8 (11.6%; 2.1%)	21 (25.9%; 5.1%)
Skin infections	3 (4.4%; 0.8%)	6 (7.4%; 1.4%)
Other^3^	18 (26.1%; 4.6%)	22 (27.2%; 4.9%)
Children of HIV-uninfected women^4^:		
N	261	258
Children with 1 or more referrals	42	39
Total number of referrals	46	45
Malaria	15 (32.6%; 5.0%)	5 (11.1%; 1.9%)
Acute diarrhea	13 (28.3%; 5.0%)	11 (24.4%; 4.3%)
Pneumonia	5 (10.9%; 1.9%)	11 (24.4%; 4.3%)
Skin infections	1 (2.2%; 0.4%)	3 (6.7%; 1.2%)
Other^3^	12 (26.1%; 4.2%)	15 (33.3%; 4.7%)
Children of HIV-infected women^4^:		
N	79	78
Children with 1 or more referrals	18	22
Total number of referrals	19	32
Malaria	6 (31.6%; 7.6%)	8 (25.0%; 9.0%)
Acute diarrhea	3 (15.8%; 3.8%)	6 (18.7%; 6.4%)
Pneumonia	3 (15.8%; 3.8%)	8 (25.0%; 7.7%)
Skin infections	1 (5.3%; 1.3%)	3 (9.4%; 2.6%)
Other^3^	6 (31.6%; 7.6%)	7 (21.9%; 7.7%)

1 Includes 137 inpatient hospital admissions and 13 (6 in the basal group and 7 in the richly-fortified group) referrals to specialist outpatient clinics.

2 Percent of children with at least one referral for each diagnosis. Note total number of referrals includes instances of more than one referral in some children.

3 Includes other infections, severe anemia, accidental injury and congenital conditions but excludes elective surgery.

4 No subgroup analyses are presented for HIV-unknown mothers because there were mixed reasons for lack of HIV testing.

**Table 7:** Hazard ratios for referral to hospital or death; richly-fortified porridge group compared to basal porridge group^1^.

	Referrals/person-yrs (rate per 100 pyrs)		HR (95% CI)	P
	Basal porridge	Richly fortified porridge		
**All children**	69/322 (21.4)	81/318 (25.5)	1.19 (0.85, 1.68)	0.31
**Stratified by:**				
Maternal HIV status^1^:				
Negative	46/219 (21.0)	45/217 (20.8)	0.99 (0.65, 1.52)	0.98
Positive	19/75 (25.5)	32/72 (44.8)	1.76 (0.95, 3.26)	0.07
**HIV-positive mothers, stratified by:**				
Child HIV status:				
Negative	14/66.2 (21.1)	14/59.7 (23.5)	1.12 (0.55, 2.29)	0.75
Positive or unknown^3^	5/8.3 (60.3)	18/11.8 (152.5)	2.45 (0.87, 6.90)	0.09
Breastfeeding duration^2^:				
<6 months	11/45.8 (24.0)	14/37.7 (37.2)	1.56 (0.70, 3.50)	0.28
≥6 months	8/28.7 (27.9)	18/33.8 (53.2)	1.92 (0.75, 4.90)	0.18
**HIV-negative children of positive mothers, stratified by:**				
Breastfeeding duration^2^:				
<6 months	7/42.2 (16.6)	8/32.6 (24.5)	1.50 (0.57, 4.00)	0.41
≥6 months	7/24.0 (29.2)	6/27.0 (22.2)	0.76 (0.27, 2.14)	0.60

1 No subgroup analyses are presented for HIV-unknown mothers because there were mixed reasons for lack of HIV testing.

2 Stratification by breastfeeding duration was done only for HIV-exposed children since this is the group for which an evidence base is required for policy.

3 HIV status could not be determined for most children who died and for those who did not complete the study; they are included in the HIV-positive or unknown group.

## Discussion

Provision from ages 6 to 18 months of a micronutrient-rich fortified porridge flour, formulated to meet all nutritional requirements according to current best estimates [9] was successful in improving children's iron status. This translated into modest improvements in linear growth amongst the subgroup of children of HIV-infected mothers who stopped breastfeeding before 6 months but had no effect on linear growth or hospital referral of the group as a whole. Among children of HIV-infected mothers, there was a non-significant trend towards increased referrals in the richly-fortified arm.

The population has clear evidence of deficiencies in iron and vitamin A, as determined in this and previous work [11]. Therefore, an effect of the richly-fortified porridge on iron status is important. It is interesting that this was achieved even though children's intake of porridge was only about half the estimate used for determining the micronutrient levels in the richly-fortified porridge. The porridge was also generally prepared more dilute than given in the instructions but, based on the pilot study for the trial [17], calories were not the limiting factor in these children's diets and increasing porridge energy density using α-amylase did not affect growth.

All children were provided with porridge and medical care which could be why the overall stunting rate was lower than the 40% [30] used in sample size estimates. This 40% rate is consistent with the 36% stunting we measured among Chilenje clinic children aged 18 months who were not part of the CIGNIS study because they reached 6 months old before the recruitment period but were otherwise similar. The risk of stunting among study children compared with clinic children was: OR 0.46; 95% CI 0.25, 0.87; P = 0.007. Therefore, effects of rich fortification, compared with basal, on stunting may have been hard to detect. This problem has been seen in other food-based intervention trials [31] in low income countries where providing the control group with food is essential both ethically and logistically, i.e. to ensure equivalent compliance between groups. Low stunting prevalence could also have resulted from biased recruitment due to a refusal rate which was higher than expected, based on our previous study in Chilenje. However, we determined that birth weights of CIGNIS children were virtually identical to those of the total population of infants born in Chilenje clinic during the same period (data not shown).

Importantly, the overall rate of hospital referral plus death did not differ between treatment groups. In the richly-fortified porridge group, there were no mortalities among infants of HIV-negative mothers. Among HIV-exposed children, there was a non-significant trend toward increased referrals in the richly-fortified group, with only pneumonia referrals increased. The subgroup analysis by maternal HIV status was planned because of its policy relevance but numbers were small; thus, the trend may be from chance and should be interpreted with caution. If not chance, possible explanatory factors include: an immune reconstitution syndrome [32] which could lead to increased respiratory symptoms; increased respiratory symptoms as have been previously observed following vitamin A supplementation [33] but not fortification; folate antagonism of the cotrimoxazole [34] which the all children of HIV-infected mothers were given as standard of care; operational issues regarding differential diagnoses resulting from improved prevention and treatment of malaria. [35] Adverse effects of iron on malaria, as reported with iron supplementation of Tanzanian children, [12] are unlikely since there were fewer malaria referrals for children given the richly-fortified porridge. Other infections may also be increased following iron supplementation of non-deficient children [12] but we are not aware of studies showing increased illness with iron fortification. Since micronutrient fortification is a key part of prevention of micronutrient deficiencies globally,[36] factors leading to hospital referral among this population are important and are under further investigation.

Although antiretroviral prophylaxis can reduce HIV transmission during early lactation [6] and although HIV-infected infants should continue breastfeeding [5], optimal feeding practises for HIV-exposed, uninfected infants over 6 months is a current concern for policy and practice. In our study, among middle class urban HIV-infected women with access to treated water and good medical care, there was no overall effect of breastfeeding after 6 months on stunting. Provision of the richly-fortified porridge improved linear growth among non-breastfed infants of HIV-infected mothers. A recently published study also from Lusaka found growth faltering, as measured by weight-for-age, among children whose HIV-infected mothers stopped breastfeeding early but no such faltering as measured by length-for-age. [37] Given the effects on linear growth we found among HIV-exposed children breastfed <6 months, it is likely relevant that we used considerably higher fortificant levels for the richly-fortified porridge than were in the maize blend cereal provided in the other study.

In summary, the rich micronutrient fortification did not reduce stunting or hospital referral but did improve iron status and reduce anemia. For the large proportion of HIV-infected mothers who stopped breastfeeding before 6 months, the richly micronutrient-fortified porridge may be a choice which could benefit their children's linear growth. However, the major effects on stunting appear to have resulted from factors, possibly improved health care, associated with participation in the study, as has been seen elsewhere [38], not from higher intake of micronutrients.

## Supporting Information

Checklist S1 CONSORT checklist.
(0.11 MB PDF)

Protocol S1 Trial Protocol.
(0.06 MB PDF)
