# Supplementary material for: Micronutrient Fortification to Improve Growth and Health of Maternally HIV-Unexposed and Exposed Zambian Infants: A Randomised Controlled Trial
Source: PLoS One. 2010 Jun 17;5(6):e11165. doi: 10.1371/journal.pone.0011165 (PMC2887362; doi:10.1371/journal.pone.0011165)
Supplement: Protocol S1 — Trial Protocol. (0.06 MB PDF) [file pone.0011165.s002.pdf]

Chilenje Infant Growth, Nutrition and Infection Study (CIGNIS) Trial Protocol

Principal Investigator: Suzanne Filteau, London School of Hygiene and Tropical Medicine (LSHTM) ; Zambian Lead Investigator: Lackson Kasonka, University Teaching Hospital (UTH), Lusaka; Senior Investigators: Rosalind Gibson, University of Otago, New Zealand; Ursula Gompels, LSHTM; Shabbar Jaffar, LSHTM; Emmanuel Kafwembe, Tropical Diseases Research Centre, Ndola; Mwaka Monze, UTH; Moses Sinkala, Catholic Relief Services, Zambia; Andrew Tomkins, Institute of Child Health, University College, London; Rodah Zulu, National Institute of Science and Industrial Research, Zambia

Trial registration # ISRCTN37460449 (www.controlled-trials.com/mrct)

Aims:

- To develop two porridges based on locally available maize and beans using: a) conventionally fortified maize, b) more richly micronutrient-fortified maize.
- To evaluate these in infants recruited at the age of 6 months and followed up for 12 months, using a randomised controlled trial. The primary endpoint will be growth. Impact will also be determined on ii**)** body composition, iii) indices of Fe, Zn, Se and vitamin A status; iv) morbidity; v) gastrointestinal permeability; vi) response to oral polio vaccine; vii) behavioural development, viii) development of chronic childhood viral infections.

Methods

A randomised controlled intervention trial will be conducted in Chilenje clinic, Lusaka, the site of our current research on maternal health and infant feeding in an area highly prevalent for HIV (32% of antenatal attenders). The government has recently scaled up HIV counselling and testing of pregnant women throughout Lusaka and most mothers of study infants will know their own status. Ethical approval with be sought from the Zambian national ethics committee and that of the London School of Hygiene and Tropical Medicine. Mothers will be approached when they bring their infants for the third DPT/oral polio vaccination around 16 weeks of age in order that we can recruit infants around the recommended time to stop exclusive breastfeeding. Vaccine coverage for DPT/oral polio in Lusaka is about 90%, and about 150/month come to Chilenje for their third DPT. The inclusion criteria for the infants at 6 months of age will be: mothers agree (a) to prepare and feed their infants for 12 months the complementary food (CF) supplied to them as a dry powder; (b) to attend the specified clinic or home visits, (c) to let their infants undergo specified urine collection and blood sampling; (d) and to permit the infants to be tested for HIV. Infants with treatable illnesses, including anemia (Hb<90 g/L), will be treated appropriately and eligible for inclusion. Exclusion criteria will be: (a) evidence of chronic disease (active TB, symptomatic HIV); (b) mother does not consent to HIV testing. Informed written consent will be obtained from all the mothers/guardians. HIV-infected mothers, especially with uninfected infants, who wish to wean abruptly to avoid the transmission risk of mixed feeding will be offered support and advice such as prior accustomising infants to a cup and spoon using expressed breast milk, and using cold compresses to reduce pain from engorged breasts. Other mothers will be encouraged to continue breastfeeding and to increase the proportion of CF in the diet as the child ages.

Trial participants will be randomised in blocks of 20 to one of the CFs. Randomisation will be done using computer-generated random numbers by a statistician not involved in the project who will keep one copy of the numerical code in a locked filing cabinet. The group number will be on the child's health card and on the food packets. The richly micronutrient-fortified and conventionally fortified maize foods will be identical in taste and colour and both the mothers and the investigators will be kept blind to the codes and treatment groups assigned. Trained

research assistants will supply the CFs to participating mothers, encourage and teach them how to prepare it during their 3-monthly visits to the clinics, and keep a register of acceptability by infants and compliance by mothers to the study regimen. In addition to maternal reports of compliance, nurses will make occasional unannounced home visits to ensure the food is being prepared and fed correctly. Participants will be encouraged to use the provided food as the main complementary food but to add items such as fruits and vegetables as desired. Extra CF will be supplied to the family according to the number of siblings under 3 years. To enhance compliance to the study protocol and completeness of follow-up, mothers will be given travel costs to the clinic.

Intervention monitoring

Anthropometry, morbidity and clinical assessments, developmental milestones, and 24-hour dietary recalls will be conducted 3-monthly as described below. Blood sampling, intestinal permeability and cognitive function tests will be conducted at 6, 12 and 18 months using established techniques (below). Immune function will be assessed at 12 months of age as acute phase protein production and antibody response to polio vaccination. Status of vitamin A and the minerals Fe, Zn, and Se will be measured in 6 and 18 month blood samples.

Socioeconomic and demographic variables

Baseline socioeconomic and demographic data will be assessed by questionnaires developed in our ongoing study in order to evaluate comparability among the treatment groups.

Anthropometry

Measurements of growth (nude weight, length, head circumference, knee-heel length) and body composition (arm circumference; triceps and subscapular skinfolds) will be made in triplicate using standardized anthropometric techniques and calibrated equipment, from which indices of body fat (arm fat area) and muscle (arm muscle area) will be calculated. We will calculate Z-scores for growth, body composition and body mass index. Birth weights will be available for most infants since about 90% of women in Lusaka deliver in clinics or hospital and record keeping is good so we will be able to calculate growth velocity since birth.

Current infant feeding

Trained research assistants will carry out a pretested modified 24-hour interactive recall (validated via a 3-day weighed record) on the mothers or caregivers to determine frequency of breast feeds and infant intake of the assigned CFs and any other CFs, breast milk substitutes, and family foods every 3 months. Median intakes and major food sources of energy and nutrients, as well as major food groups (in grams) will be determined from the recalls using food composition values compiled from the World Food Minilist, plus additional analyzed values for trace minerals and phytate from Malawian food composition values. Estimates of nutrient adequacy from CFs for each group will be determined by comparing intakes (per day, per 100 kcal) with corresponding estimated nutrient needs and desirable nutrient densities. Nutrient intakes will be compared with WHO requirement estimates.

Morbidity

It is likely that only moderate-severe morbidity makes a detectable impact on nutritional status and that such morbidity will be better remembered than mild morbidity. However, current morbidity at the time of visit will affect current infant diet. Therefore, we will collect two types of infant/child morbidity data by questionnaire: 1) overall infant morbidity in the last 3 months (scored as healthy; mild, self-limited illness; moderate illness requiring symptomatic treatment at the clinic; severe illness requiring antibiotics or other medical intervention); and 2) symptoms (diarrhea, lower respiratory infection, ear suppuration, skin infections, purulent conjunctivitis,

oral thrush) within the past 3 days, an interval shown to provide adequate recall data for diarrhea. These two methods provided overlapping and consistent information in our previous work in South Africa. In addition, project nurses will examine the infants at routine project visits and mothers will be encouraged to bring their infants to these nurses whenever the infants are ill. Finally, we will measure the acute phase proteins, C-reactive protein (CRP) and α_1-acid glycoprotein (AGP), since acute phase proteins have been shown to correlate with maternal recall of infant symptoms.

Since maternal illness, especially among HIV-infected mothers, may affect child care and feeding of the CFs, at all visits we will collect the two types of questionnaire data above for maternal as well as child health. Mothers will be encouraged to come to the clinic when they themselves are ill in order to obtain treatment from project nurses or doctors.

<u>Micronutrient status</u>
Vitamin A and Fe status were chosen for assessment because deficiencies are common and serious worldwide, there is evidence of deficiency in Zambian infants, and vitamin A status is especially affected by both inadequate maternal status and low breast milk intakes. Zn was selected because its bioavailability from local maize-based CFs is low and there is also antagonism at the level of intestinal absorption between Zn and Fe. In urban Chilenje, Lusaka, which is better off than rural Zambia and where infants are likely growing faster, Zn may become limiting for infant growth which it did not appear to be in rural Zambia. Se will be measured because of evidence that deficiency can impair immune function and increase virulence of some viruses; there is no information about Se status of Zambians.

Whole anticoagulated blood will be used to measure hemoglobin by Hemocue in the clinic. Veni-puncture blood samples will be collected in trace-element free evacuated containers (Monovette Sarstedt) using standardized conditions, as specified by the International Zinc Nutrition Consultative Group, refrigerated immediately, and sent for serum separation within 4 hours in the UTH lab. Care will be taken to use trace element-free polyethylene vials and transfer pipets and powder-free gloves. Serum will be frozen at -80°C and taken in batches on dry ice to the WHO Tropical Diseases Research Centre, Ndola, for analysis. Zn and Se will be analysed by atomic absorption spectroscopy, retinol by HPLC; CRP, ferritin and transferrin receptor by ELISA, and AGP by turbidimetry.

<u>Permeability tests</u>
Increased gut permeability is associated with infection, poor micronutrient status and decreased weight gain. Lactulose/mannitol dual sugar permeability tests will be conducted as described previously. Children are given a measured dose of the two non-metabolisable sugars and urine is collected for 5 hours. The technique has worked well in South Africa with good compliance for tests on young infants and was sufficiently sensitive to measure differences between healthy breastfed and non-breastfed infants and between HIV-infected and uninfected infants. Mothers will be given a meal in the clinic during the 5 hour infant urine collection.

<u>Immunological and virological assessment</u>
Deficiencies of both macro- and micro-nutrients impair immune functions. We will investigate three aspects of immunity to viruses which have major impacts on child health: i) response to polio vaccine, ii) human herpesvirus 6 (HHV-6) infection, iii) prevalence of chronic human cytomegalovirus (HCMV) infection. For all responses the effect of HIV seropositivity will be examined. The lab work will be conducted in the virology departments of UTH and the LSHTM.

Polio: The response to oral polio vaccine, both acutely and in the longer term, was selected because it is safe, relevant to child health, and involves the gut epithelial immune response (particularly sensitive to nutrition), as well as inducing a systemic antibody response. Furthermore, the response to polio has been shown to be higher in breastfed than non-breastfed European and American infants. A recent survey of coverage and immunogenicity of oral trivalent polio vaccine in Zambian children under 5 years showed that about 85% of children had protective titres after the standard three doses in early infancy but that it was recommended that a booster be given in later infancy in order to induce protective titres in all children and ensure high coverage. The researchers recommended giving the booster at the 9 month measles vaccine for convenience, although this has yet to become policy. We will delay until 12 months, which will be unlikely to result in additional risk to children, in order to avoid immunological complications of the measles vaccine and to permit 6 months' consumption of the intervention diets before vaccination.

Children will have fingerprick blood samples taken for measurement of CRP and of baseline polio titre by plaque neutralisation. Children will be given the standard dose of oral trivalent polio. Repeat fingerprick blood samples will be taken at a home visit 24 hours later to measure CRP as an indicator of the acute phase response to the vaccine. The venous blood sample at 18 months will be used to measure post-immunisation titre.

HHV-6 has been identified in this population and can also contribute to transient immunosuppression. Cases of unexplained fever will be analysed for HHV-6 DNA by specific PCR on serum to detect active replication.

Chronic HCMV infection: HCMV presents serious risks to Zambian children and establishes a lifelong persistent infection. If there are immune deficiencies or aberrations, including lack of protective maternal immunity, the primary pediatric infection can lead to fatal pneumonitis as well as neurological damage including retardation or effects on hearing. Although auditory effects will not be directly monitored in this study, indirect effects on speech and motor development will be followed (see below). The most severe risks are from early infection: congenital, perinatal or postnatal through breast milk or saliva. While the exact time or route of infection will not be monitored here, overall these infections can be identified. In HIV-1 infected American infants, HCMV infection acquired before 18 months had greater CNS disease and disease progression leading to mortality then HIV-1 alone. In Zambia, fatal pneumonitis in pediatric HIV/AIDS due to HCMV infection was a main infectious cause of death, equivalent or in some cases higher than due to tuberculosis. In adults HCMV DNA has been shown as a better predictor of mortality than a high HIV viral load.

DNA will be extracted from blood samples at 6, 12 and 18 months using microextraction kits (Qiagen) as described previously. PCR will use specific primers which can determine strain identifiers based on hypervariable glycoprotein genes (gN/gO) which we have shown are linked and mediate infection by the virus. Recent results further suggest a trend linking some genotypes with pediatric infection or HIV/AIDS and our initial results in Zambia have identified these genotypes. Qualititative PCR and sequence analysis (ABI) followed by phylogenetic analyses for strain genotype will be as described previously. Quantitative PCR to determine levels of viremia will be performed on selected samples using a COBAS Amplicor CMV Monitor system (Roche Diagnostic Systems) as described or using Taqman technologies (PerkinElmer). Serological assays using commercial diagnostic ELISA kits will be performed on sera isolated at 18 months to confirm extent of HCMV infection in the children.

HIV testing: Infection with HIV is routinely diagnosed at UTH using commercial serological assays (Bionor or EIA, Organon Teknika).

Behavioural development
Chronic undernutrition, as indicated by stunting, as well as deficiencies of micronutrients such as Fe and Zn, can impair behavioral development. Furthermore, immunosuppression from malnutrition may lead to more severe primary infection with HCMV which can cause neurological abnormalities including retardation, hearing impairment and retinitis, which may affect behavioural development. At the 3-monthly clinic visits, trained research assistants will interview all the mothers using a structured questionnaire to determine whether the following specific developmental milestones had been achieved: motor skills eg. creeping, standing with support, standing unsupported, walking supported; language skills eg. babbles, says 3-4 clear words; social skills eg. smiles when smiled at, shows apprehension at strangers. Prior pilotting will determine suitability of the items, and interobserver and test-retest reliabilities.

Developmental quotients will be assessed at 18 months using the Motor and Mental Scales of the Bayley Scales of Infant Development (BSID-II; The Psychological Corporation). During the test, the testers will observe the children's behaviour and record the following on 9 point rating scales: activity, vocalisations, affect, response to examiner, cooperation with test procedure, attentiveness and task orientation. These ratings have been used previously in less-developed countries and were reliable and sensitive. The tests will be carried out in a dedicated test room at the clinic. The testers will be trained beforehand until acceptable levels of interobserver reliabilities are achieved. Before the data collection is started, the test will be pilotted among Zambian children of the same age range and small modifications may be made to make it more culturally appropriate.

Data analysis and sample size calculations
Primary analyses will be by intention-to-treat but analyses according to compliance with the feeding protocol will also be done. The proportion of children stunted (height for age Z<-2 SD) will be compared between groups using the Chi-squared test while mean anthropometric Z-scores will be compared using a t-test. Analysis adjusting for HIV-status fitted as a covariate will be conducted. The multivariate analysis will be by unconditional logistic regression for categorical variables and by analysis of variance for continuous variables.

We estimate 150 mother-infant pairs will be eligible for recruitment each month, making the total eligible 1800 in the year. Based on our previous studies in the area, we assume no more than 20% will refuse to enter the trial, primarily due to refusal of HIV testing, and 15% will be lost to follow-up, leaving 408/group for analyses. Assuming that 40% of children will be stunted in the conventional maize group, the trial will have 93% power to detect as statistically significantly a proportion stunted of 28% (a 30% reduction in the proportion stunted) in either of the intervention arms and 83% power to detect a proportion stunted of 30% (a 25% reduction in the proportion stunted).

The sample size of 408/group will be adequate to detect biologically relevant differences in key secondary endpoints also. At 90% power and based on micronutrient status data from Zambian children, we will need only 165 children/group to detect a 5 g/L difference in hemoglobin, the smallest change of any biological relevance, and 182 per group to detect a change in proportion of children with deficient (< 0.7 μmol/L) plasma retinol from 50% to 35%. For polio titre, about 10% of Zambian children have inadequate titres after 3 doses of vaccine and to improve this to 3% will require 286 children per group. A difference of 6 developmental quotient points has been found in studies of nutritional supplementation in Jamaica and is probably the lower limit of

functionally important differences. Assuming a difference of 6 points between the groups and a standard deviation of 10 points from previous work in Jamaica, a sample size of 58/group is required for 90% power.
